# Prospective quantitative study: Doppler ultrasound in the evaluation of chronic renal allograft disease and correlation with histopathological finding

**DOI:** 10.1097/MS9.0000000000001251

**Published:** 2023-09-05

**Authors:** Shailendra Katwal, Sundar Suwal, Rajan M. Bhandari, Dinesh Chataut, Mukhtar Alam Ansari, Suman Lamichhane

**Affiliations:** a Dadeldhura Subregional Hospital, Dadeldhura; b Tribhuvan University Teaching Hospital, Maharajgunj; c Nepal A.P.F Hospital, Balambu, Kathmandu, Nepal; d Postgraduate Institute of Medical Education and Research, Chandigarh, India

**Keywords:** chronic allograft nephropathy, doppler ultrasonography, fibrosis, resistive index

## Abstract

**Background and Objectives:**

Renal allograft biopsy is the gold standard for diagnosing chronic allograft nephropathy, but noninvasive methods are needed to avoid unnecessary biopsies. Doppler ultrasonography, particularly the resistive index (RI), correlates with renal allograft dysfunction. This study aims to assess the relationship between renal sonographic parameters and biochemical parameters in diagnosing graft interstitial fibrosis.

**Methods:**

The study evaluated 60 renal allograft recipients for sonographic renal morphological features and Doppler indices. The estimated glomerular filtration rate (eGFR) was calculated, and cortical fibrosis after the biopsy was determined using the Banff score. Continuous variables like mean and SD were calculated, and categorical variables were reported using frequencies and proportions. Associations were examined using independent sample *t*-tests, *χ* ^2^tests, and multivariate regression analysis.

**Results:**

The mean eGFR was 75.23±25.45 ml/min/1.73 m^2^. A significant correlation of eGFR with RI (*r*=0.341, *P*=0.008) was seen. A significant difference in mean RI (F=10.167; df=2,57; *P*<0.001) was seen among the histological grades of fibrosis. Among the histological grades of fibrosis, significant differences in RI among mild and moderate (S.E. 0.033, *P*=0.043), mild and severe (S.E. 0.026, *P*=0.001) as well as moderate and severe (S.E. 0.036, *P*=0.029) was seen.

**Conclusion:**

Doppler was able to noninvasively predict allograft fibrosis and could be used as a complementary imaging tool during the follow-up of renal allograft patients. Future research is needed to improve evidence, diagnostic criteria, guidelines, and long-term impact.

## Introduction

Highlights

- Doppler ultrasound particularly resistance index is a reliable noninvasive tool for diagnosing and monitoring renal allograft disease.
- Doppler ultrasound provides valuable insight into renal function in renal allograft recipients.
- Doppler ultrasound can reduce the need for invasive biopsies, personalizing the treatment approaches, and improving patient care and outcomes.

The leading cause of chronic allograft failure is chronic allograft dysfunction among renal transplant recipients, despite the significant reduction in acute rejection rates over the last decades^1^. The progressive decline in renal function is detected biochemically by an increase in the level of serum creatinine; however, the rate of increase in serum creatinine lags behind the course of chronic allograft dysfunction and occurs late in the course of the disease^2,3^. Thus, relying on the level of serum creatinine to identify the transplant recipients at risk, can delay the measures to retard or prevent the development of subsequent graft loss^4^. A biopsy can diagnose chronic allograft dysfunction. However, it is an invasive method prone to many complications, including graft loss^5^.

Ultrasonography is a safe and noninvasive modality that can assess renal parenchymal echogenicity, morphology, and vascular resistance, which can predict the progression of chronic allograft dysfunction. Peak systolic velocity (PSV) and renal arterial resistive index (RI) are important Doppler parameters to assess for renal arterial disease. Kidney rejection causes decreased blood perfusion and increased resistance in the renal vasculature, leading to alterations in blood flow velocity during diastole. This results in reduced diastolic flow, which serves as an indicator of a poor prognosis for graft survival^6^. Power Doppler imaging plays a crucial role in renal transplantation, providing noninvasive insights into blood flow patterns. It has better sensitivity than conventional color Doppler imaging in detecting small peripheral vessels and normal renal vasculature. The technique’s sensitivity to low flow facilitates the visualization of perfusion patterns, distinguishing rejection from other causes of graft dysfunction. Serial power Doppler examinations offer valuable follow-up monitoring, complementing traditional biopsy-based diagnosis^7^.

Limited literature is available to study the role of sonography in chronic allograft rejection. Thus, this study aimed to establish correlations between the histopathological findings and the biochemical and sonographic morphological as well as Doppler parameters, which can help clinicians understand the disease pathology more accurately and assess and monitor the status of transplant kidneys based on noninvasive sonographic reports.

## Methodology

**Figure 1:**
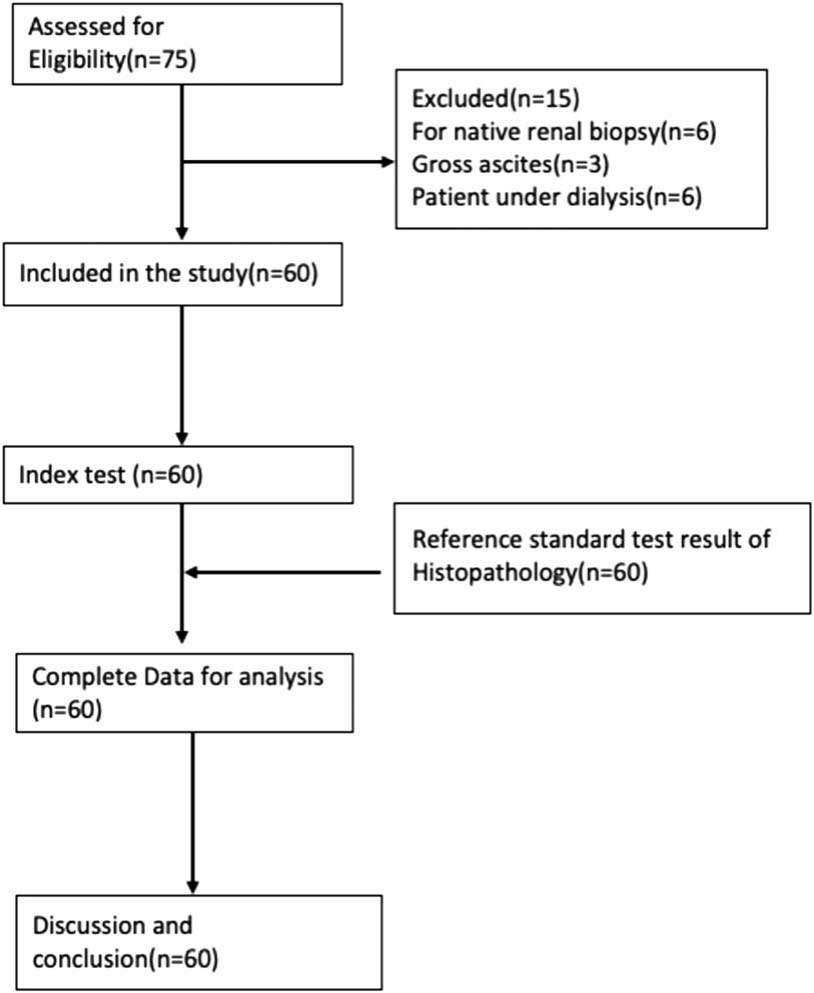
STROCSS flow diagram of the participant.

This study was a prospective quantitative study conducted on 60 patients referred to the Department of Radiology and Imaging for renal allograft biopsy between November 2018 and November 2019. The sample size was determined based on findings from a similar study conducted by Griffin *et al*.^8^. Ethical clearance for the study was obtained from the Institutional Review Board, reference number (28(6-11-E)2/075/076), and informed written consent was obtained from all participating patients after explaining the study to them. Out of the selected 75 patients, individuals presenting for native renal biopsy, those with gross ascites, and patients under dialysis were excluded, resulting in a final sample size of 60 patients. Clinical and laboratory parameters were recorded using a pre-designed proforma, and all eligible patients underwent ultrasonographic examination following the standard protocol of the department (Fig. 1). This study followed the STROCSS (Strengthening the Reporting of Cohort Studies in Surgery) 2021 checklist for cross-sectional studies^9^. The study is registered retrospectively in the research registry with a unique identification number (UIN) of researchregistry9208 https://www.researchregistry.com/browse-theregistry#home/registrationdetails/649dcad76164b70028dd23e8/.

**Figure 2:**
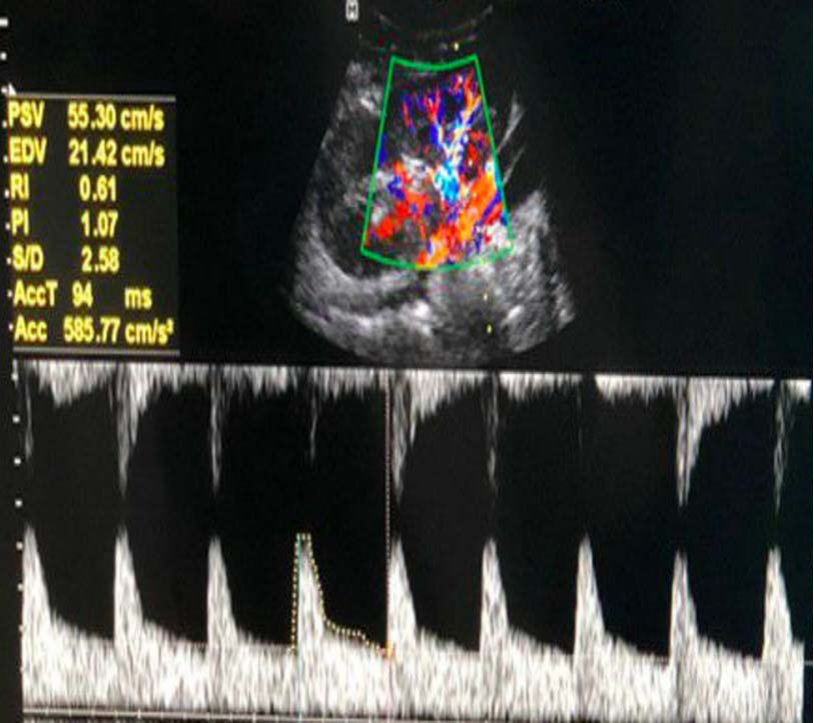
Spectral Doppler study showing the measurement of Peak systolic velocity (PSV) and Resistance index (RI).

The study involved an ultrasound examination of the kidney, including morphologic features such as length, echogenicity, corticomedullary differentiation, and cortical thickness. Renal cortical thickness was measured in the sagittal plane over a medullary pyramid, and renal length was measured in the mid-sagittal plane. Intrarenal color Doppler analysis was performed at three representative locations in the kidney, with a pulse repetition frequency between 700–1000 MHz, a wall filter, and appropriate color gain. Peak systolic velocity and resistance index were measured at the upper, middle, and lower poles, and the average value was recorded. The average of these measurements was used for statistical analysis (Fig. 2).

After diagnostic ultrasonography, preintervention steps involved patient preparation, medical history assessment, coagulation status evaluation, contraindication identification, and bleeding risk assessment. Patient comfort and emotional well-being were prioritized, ensuring a smooth and effective intervention. The biopsy was performed under USG guidance using a core biopsy needle and sent for histopathological examination. Allograft biopsies were performed by specialized interventional radiology operators with significant experience, averaging 68 procedures annually, demonstrating proficiency and familiarity with the biopsy technique. A standardized protocol for allograft biopsy was developed, followed by operators, including patient preparation, technique, and postprocedure care. Regular meetings and continuous monitoring were done for quality control. After a biopsy, patients were advised to avoid heavy lifting, care for wounds, report infections promptly, and schedule follow-up appointments to monitor recovery progress and potential complications. The histopathological reports were followed, and the Banff grading of the allograft interstitial fibrosis^10^ was obtained and correlated.

The data were recorded in Microsoft Excel (2016), and analysis was done with Microsoft Excel as well as SPSS (Version 22). The mean and SD were calculated for continuous variables. Categorical variables were reported using frequencies and proportions. The association between variables was examined using an independent sample *t*-test and *χ* ^2^test. Multivariate regression analysis was also performed to measure the association among variables.

The study involved patients and the public in the research objectives, design, and outcome measures. They facilitated recruitment, provided feedback, and participated in meetings, dissemination, and knowledge translation activities.

## Results

The study included 60 patients within the age range of 18 to 59 years, consisting of both males and females. The mean age of the patients was 35.97 years (Table 1).

**Table 1:** Clinico-demographic profile of allograft recipients referred to radiology department.

Characteristics	Number (%)
Sex	
Male	8 (13.33%)
Female	52 (86.67%)
Age	
>60 year	0
50–59 year	6 (10%)
40–49 year	15 (25%)
30–39 year	20 (33.33%)
<30 year	19 (31.67%)

Among 60 patients, the mean eGFR was 75.23±25.45 ml/min/1.73 m^2^ (male - 76.13±24.77 ml/min/1.73 m^2^ and female - 69.37±30.70 ml/min/1.73 m^2^). Eight (13.3%) patients presented with an eGFR of less than 50 ml/min/1.73 m^2^, five males and three females.

The mean allograft renal length was 10.10±0.89 cm (male – 10.13±0.88 cm and female - 9.93±0.97 cm). Normal parenchymal echotexture was seen in 24 patients. Among 36 patients with increased renal parenchymal echotexture, 22 patients had Grade I, 10 had Grade II, and four had Grade III increased renal parenchymal echogenicity. Corticomedullary differentiation was maintained in 46 (76.7%) patients, poorly maintained in 11 (18.3%) patients, and not maintained in three (5%) patients.

All the renal allografts were seen to have at least some grade of fibrosis and were categorized into one of the three classes as per the Banff score. Most of the patients (43–71.7%) had a mild degree of fibrosis (<25%). Thirteen (21.7%) and four (6.7%) patients had moderate (25–50%) and severe (>50%) fibrosis, respectively.

The mean renal lengths in the eGFR categories greater than 50 ml/min/1.73 m^2^ and <50 ml/min/1.73 m^2^ were 10.25±0.83 cm and 9.12±0.52 cm, respectively. There was a significant, moderately positive correlation between eGFR and renal length (*r*=0.341, *P*=0.008). Also, there was a significant difference in mean renal length among the two groups with eGFR less than 50 and greater than 50 ml/min/1.73 m^2^ (t= −3.674, df=58, *P*=0.001) (Table 2).

**Table 2:** Correlation of EGFR with renal length (*N*=60).

								95% CI	
	EGFR (ml/min/1.73 m^2^)	*N*	Mean	SD	*t*	df	Sig. (2-tailed)	Lower	Upper
Renal_ Length	less than 50	8	9.125	0.5230					
	more than 50	52	10.253	0.8399	–3.674	58	.001	–1.7421	–0.5132
Cortical thickness (mm)	less than 50	8	7.825	0.9392					
	more than 50	52	10.862	9.5353	–0.894	58	0.375	–9.8383	3.7653

The mean cortical thickness of the sample was 10.45±8.9 mm. The mean cortical thickness in the two eGFR categories greater than 50 and less than 50 ml/min/1.73 m^2^ were 10.86±9.53 mm and 7.82±0.93 mm, respectively. However, no significant correlation was seen between cortical thickness and eGFR (*r*=0.011, *P*=0.93). No difference in mean cortical thickness was seen among the two eGFR categories (t= −0.894, df=58, *P*=0.375) (Table 2).

The mean PSV and the mean RI of the main renal artery were 45.89±12.75 cm/s and 0.74±0.11, respectively. The range of PSV was 20.3 to 78.3 cm/s and that of RI was 0.5 to 0.94. There was a significant moderately negative correlation between the mean RI and the mean renal size (*r*=−0.397, *P*=0.002). However, there was no significant correlation between PSV and renal size (*r*=0.040, *P*=0.310). There was no significant correlation between PSV and RI with the cortical thickness (*P*=0.597 and 0.255) respectively (Table 3). There was no significant correlation between PSV and RI with eGFR (*P*=0.124 and *P*=0.400, respectively).

**Table 3:** Correlation of peak systolic velocity (PSV) and resistive index (RI) with renal length and cortical thickness (*n*=60).

Variables	Mean	SD		PSV	RI	RL (cm)	CT (mm)
PSV (cm/s)	45.89	12.75	Pearson correlation	1	0.150	0.040	−0.078
			Sig. (2-tailed)		1.15	0.31	0.597
			*N*	60	60	60	60
RI	0.7370	0.11060	Pearson correlation	0.150	1	–0.397	0.033
			Sig. (2-tailed)	1.15		0.002	0.255
			*N*	60	60	60	60
RL (cm)	10.102	0.8897	Pearson correlation	0.040	−0.397	1	0.090
			Sig. (2-tailed)	0.31	0.002		0.496
			*N*	60	60	60	60
CT (mm)	10.457	8.9320	Pearson correlation	−0.078	0.033	0.090	1
			Sig. (2-tailed)	0.597	0.255	0.496	
			N	60	60	60	60

Among the 43 patients with mild fibrosis, most of the patients (*n*=23, 53.49%) had normal RI. Only four of them had RI greater than 0.8. All the patients with severe fibrosis had RI greater than 0.8 and most of the patients with moderate fibrosis (*n*=7, 53.85%) also had RI greater than 0.8. Only two patients (15.38%) with moderate fibrosis had normal RI (<0.7). Thus, there was a significant difference in mean RI among the histological grades of fibrosis (F=10.17; df=2,57; *P*<0.001) (Table 4). Among the histological grades of fibrosis, there was a significant difference in RI among mild and moderate (S.E. 0.033, *P*=0.043), mild and severe (S.E. 0.026, *P*=0.001) as well as moderate and severe (S.E. 0.036, *P*=0.029). The mean RI was found to be higher in higher grades of fibrosis.

**Table 4:** Correlation of resistive Index (RI) with histological grades (*n*=60).

Histological grades	*N*	Mean RI	SD	Variance	Sum of squares	df	*F*	Sig.
Mild fibrosis (<25%)	43	0.7051	0.09615	Between grades of fibrosis	0.190	2	10.17	0.000
Moderate fibrosis (25–50%)	13	0.7923	0.10725					
				Within grades of fibrosis	0.532	57		
severe fibrosis (>50%)	4	0.9000	0.04320					
Total	60	0.7370	0.11060		0.722			

## Discussion

Ultrasonography with Doppler study is the most common imaging modality for the routine evaluation of the renal length, cortical thickness, parenchymal echotexture, and any other abnormalities in renal allograft recipients. However, a single parameter cannot predict the exact in vivo condition of the renal allograft. Among various sonographic parameters, the RI has been widely used to reflect the status of renal parenchyma despite its poor specificity^11^. Normal RI in allograft kidney is less than 0.7; if RI is greater than 0.8, it is abnormal. RI between 0.7 and 0.8 is considered indeterminate. An RI cutoff of 0.8 demonstrated a sensitivity of 38% and a specificity of 63%. Additionally, there was no significant difference in the RI values between acute and chronic allograft dysfunction cases^12^.

Renal allograft biopsy, though invasive, is the recognized diagnostic method for the evaluation and management of the allograft, including the determination of its prognosis and viability. Due to its invasive nature, the allograft biopsy procedure can have various complications in 12–13%, the most common being hematuria^5^.

In our study, the mean allograft renal length was 10.10±0.89 cm (male – 10.13±0.88 cm and female - 9.93±0.97 cm). A significant moderate positive correlation between eGFR and renal length (*r*=0.341, *P*=0.008) was seen in our study. However, no statistically significant correlation was observed between kidney volume and eGFR in the study done by He WY *et al*.^13^. This discordance might be because the latter used renal volume rather than renal length for the estimation of renal size.

Our study showed no correlation between the renal allograft cortical thickness and the eGFR. However, a study done by Hoi Shotaro *et al*.^14^ found a correlation between renal cortical thickness and eGFR (*r*=0.426, *P*<0.001) but this study was done in the native kidneys, and the literature regarding cortical thickness and eGFR in renal allografts remains to be evaluated with further studies in the future.

In our study, a significant, moderately negative correlation was seen between the mean RI and the mean renal size. But, no statistically significant correlation was observed between kidney size and RI in the study done by He Wy *et al*.^13^. However, they used renal volume instead of renal length, unlike our study for the estimation of renal size.

Biopsy showed fibrosis in all the patients in our study; however, most (71.7%) had a mild degree of fibrosis, and only 6.7% of patients had severe (>50%) fibrosis. In our study, a significant difference in mean RI among the histological grades of fibrosis (F=10.167; df=2,57; *P*<0.001) was seen in our study. Orrlachio *et al*.^15^ found a variable degree of increase in RI in all patients. They found out that patients with mild or moderate fibrosis had an RI lower than patients with severe fibrosis ((0.76±0.03 vs. 0.92±0.04; *P*≤0.05), which was a similar finding as in our study.

The main objective of the present study was to determine the diagnostic performance of Doppler ultrasound in chronic allograft dysfunction. As RI showed a significant correlation with histological grades of renal allograft fibrosis, noninvasive and cost-effective ultrasonography with the Doppler study can be a good adjunct in the routine evaluation of renal allografts. The study’s success is attributed to the generous contributions of patients and public representatives.

The study’s empirical results have limitations, including a smaller sample size (*N*=60) and the lack of evaluation of renal fibrosis, an ongoing process with increasing trends. A larger sample size would improve precision.

Future research should enhance evidence, refine diagnostic criteria, establish clinical guidelines, and evaluate the long-term impact of Doppler ultrasound in renal allograft disease management.

## Conclusion

There was a significant correlation between renal length with eGFR and the RI of the allograft kidney. A significant correlation of the RI is also seen with the histological grades of fibrosis. Though biopsy has been routinely used for the assessment of chronic allograft dysfunction, noninvasive ultrasonography with Doppler can also predict allograft dysfunction without any complications related to biopsy. It can also be helpful when a biopsy cannot be performed.

## Ethical approval

We have conducted an ethical approval base on the Declaration of Helsinki with registration research at the Institutional Review Committee (IRC) of the Institute of Medicine (IOM), Tribhuvan University, Nepal, Reference number: 28(6-11-E)^2^/075/076).

## Consent

Written informed consent was obtained from the patient for the publication of this case report and the accompanying images. A copy of the written consent is available for review by the Editor-in-Chief of this journal on request.

## Sources of funding

The authors declare that writing and publishing this manuscript was not funded by any organization.

## Author contribution

S.K.: conceptualization, as mentor and reviewer for this original article and for data interpretation; S.S.: conceptualization and reviewer for this case; R.M.B.: contributed in performing literature review and editing; D.C.: reviewer and data interpretation; M.A.A.: contributed in performing literature review and editing; S.L.: contributed in writing the paper and reviewer for this case. All authors have read and approved the manuscript.

## Research registration unique identifying number (UIN)

1. Name of the registry: Research Registry.
2. Unique identifying number or registration ID: Researchregistry9208.
3. Hyperlink to your specific registration (must be publicly accessible and will be checked): https://www.researchregistry.com/register-now#home/registrationdetails/649dcad76164b70028dd23e8/

## Guarantor

Shailendra Katwal is the person in charge of the publication of our manuscript.

## Provinence and peer review

Not commissioned, externally peer-reviewed.

## Data availability statement

The materials datasets used and/or analyzed during this study are available from the corresponding author upon reasonable request.
